# Convergent evolution of plant and animal embryo defences by hyperstable non-digestible storage proteins

**DOI:** 10.1038/s41598-017-16185-9

**Published:** 2017-11-20

**Authors:** María Yanina Pasquevich, Marcos Sebastián Dreon, Jian-Wen Qiu, Huawei Mu, Horacio Heras

**Affiliations:** 1 Instituto de Investigaciones Bioquímicas de La Plata (INIBIOLP), Universidad Nacional de La Plata (UNLP) — CONICET CCT-La Plata, La Plata, Argentina; 2 Cátedra de Bioquímica y Biología Molecular, Facultad de Ciencias Médicas, UNLP, Argentina; 3 Department of Biology, Hong Kong Baptist University, Hong Kong, P.R. China; 4 Cátedra de Química Biológica, Facultad de Ciencias Naturales y Museo, UNLP, Argentina

## Abstract

Plants have evolved sophisticated embryo defences by kinetically-stable non-digestible storage proteins that lower the nutritional value of seeds, a strategy that have not been reported in animals. To further understand antinutritive defences in animals, we analysed PmPV1, massively accumulated in the eggs of the gastropod *Pomacea maculata*, focusing on how its structure and structural stability features affected its capacity to withstand passage through predator guts. The native protein withstands >50 min boiling and resists the denaturing detergent sodium dodecyl sulphate (SDS), indicating an unusually high structural stability (i.e., kinetic stability). PmPV1 is highly resistant to *in vitro* proteinase digestion and displays structural stability between pH 2.0–12.0 and 25–85 °C. Furthermore, PmPV1 withstands *in vitro* and mice digestion and is recovered unchanged in faeces, supporting an antinutritive defensive function. Subunit sequence similarities suggest a common origin and tolerance to mutations. This is the first known animal genus that, like plant seeds, lowers the nutritional value of eggs by kinetically-stable non-digestible storage proteins that survive the gut of predators unaffected. The selective pressure of the harsh gastrointestinal environment would have favoured their appearance, extending by convergent evolution the presence of plant-like hyperstable antinutritive proteins to unattended reproductive stages in animals.

## Introduction

Plants have evolved a wide array of proteins to defend their embryos against herbivores, including dietary lectins, inhibitors of digestive proteases and non-digestible storage proteins which are massively accumulated in seeds^1–5^. The digestibility of these plant seed storage proteins is related to an unusually high structural stability. The biophysical basis of this relationship has been very recently ascribed to kinetically stable proteins (KSP)^6^. A similar protein-based defensive system of lectins, protease inhibitors and non-digestible proteins has not been recognized in animals, except for the aposematic egg clutches of the freshwater snail *Pomacea canaliculata* (Lamarck, 1822)^7,8^ and partially in foam nests of the túngara frog *Engystomops pustulosus* (Lynch, 1970)^9^. This is surprising given that plant and animal embryos are often exposed to similar selective pressures by predators and pathogens alike.

*Pomacea* spp. (Caenogastropoda: Ampullariidae), commonly called apple snails, are aquatic organisms that have acquired the ability to deposit brightly coloured egg clutches above water level^10^, a transition that seldom occurs in the animal kingdom. The presence of well-defended eggs in this species is reflected in the fact that few predators have been reported for *Pomacea* eggs^11^. Moreover, field studies have shown that rodents are common apple snail predators that systematically avoid eating *Pomacea* eggs and the albumen gland, which synthesizes and stores the egg perivitelline proteins^12^.

Like most gastropods, *Pomacea* egg capsules are filled with a perivitelline fluid, which surrounds the developing embryo and provides a rich source of energy and nutrients. However, recent findings have shown that they seem to have acquired a novel set of proteins^13^ that enable eggs to develop under harsh aerial conditions exposed to desiccation, often high temperatures, and terrestrial predators.

Thus far, only a few gene products have been shown to play a direct role in embryo defence against biotic and abiotic stressors in *P. canaliculata*, namely the carotenoprotein PcOvo, with a putative antinutritive role, and PcPV2, a neurotoxic lectin lethal to rodents^7,8,14–18^.

PcOvo is a storage protein, which is massively accumulated in the egg (up to 70% of the egg protein)^19^, which is consumed during embryo development providing energy and amino acids for the embryo^20^. The conspicuous pigmentation of egg masses is also supplied by PcOvo, not only protecting embryos against solar radiation and oxidative damage^14^, but also providing a warning colouration (aposematic) to deter visual-hunting predators^10,15^ rendering this apple snail probably the best example of an animal with aposematic eggs^21^. Moreover, this multifunctional macromolecule displays high thermal and pH structural stability, resistance to proteases and decreased rat growth rate when ingested, indicating PcOvo may have an antinutritive role, thereby lowering the nutritional value of eggs^7,22^. It is interesting to note that PcOvo subunits, likely paralogues themselves, show no similarity with any known sequence^13^.

Biochemical analysis of another apple snail egg carotenoprotein, PsSC from *Pomacea scalaris* (d´Orbigny, 1835) showed that although they share several structural and functional properties, only PsSC displays strong lectin activity^23,24^. This provided evidence that these protective perivitellins have undergone a rapid evolution in closely related species^24^.

In the present study, we focus on PmPV1, the egg storage protein of *Pomacea maculata* Perry, 1810^25^, a worldwide invasive species, a major pest of aquatic crops^26^ and an intermediate host of a human parasitosis^27^. PmPV1 is an oligomeric protein that shares biochemical similarities with PcOvo^25^, and is the most abundant egg perivitellin (~64% of the egg protein^28^). Thus, PmPV1 is also involved in the protection of carotenoids until they are taken up by the embryo, whose carotenoid content plays a role in photoprotection, conspicuous egg colouration and antioxidative defence for the developing embryo, similar to PcOvo^14,16^.

Plant and animal embryos are often exposed to similar selective pressures by predators and pathogens alike, as both animal eggs and plant seeds are highly concentrated nutrient sources. This has resulted in the development of KSP in plant seeds, which has been related to a non-digestible defence against predation^6^. This has not been studied in animals.

Considering that dietary protein quality has a significant impact on predator-prey relations^29^, and to further understand the potential antinutritive snail egg defence against predators, we studied the structure-function relationship of PmPV1 carotenoprotein. This multifunctional perivitellin participates in nutrition, energy supply and putative defence, which makes it an attractive subject for research in animal proteins that exert antinutritive effects on predators. We report its low-resolution 3D model, its phylogenetic relationships, the structural perturbation by physical and chemical agents, its kinetic stability and a remarkable capacity to withstand the gastrointestinal environment of a potential predator. We discuss the importance of its high kinetic stability and non-digestible properties on its putative function in egg defence against predation and above water environmental conditions.

## Results

### Structure

#### Subunit sequences

A search in *P. maculata* albumen gland transcriptome using N-terminal sequences of PmPV1 subunits allowed us to identify four subunits. An additional search using these subunits identified two more potential subunits in the transcriptome. The 6 sequences contained 196–210 translated residues, the first 18–21 amino acids of which were a putative secretory pathway signal peptide (Supplementary Fig. 1a). After removing the signal peptide, the theoretical molecular mass of the mature subunits ranged between 19.8–21.6 kDa, in agreement with the experimental MW obtained for the subunits by PAGE after deglycosylation (24 kDa)^25^. The six subunits displayed predicted phosphorylation and glycosylation sites. Eighteen phosphorylation sites were predicted to be associated with tyrosine (nine sites), serine (three sites), and threonine (six sites) in PmPV1 subunits (Supplementary Fig. 1a). Three subunits displayed a single predicted N-link glycosylation site (NXS/T), while one of them (PmPV1-1) had two more predicted N-glycosylation sites (NXS/T) with low probability of being glycosylated. Two N-linked glycosylation sites (NXS/T) were predicted in the potential subunit PmPV1-5 and three in PmPV1-2 and the potential subunit PmPV1-6 (Supplementary Fig. 1a). The full cDNA sequences reported here are deposited in GenBank (www.ncbi.nlm.nih.gov/genbank/) with accession numbers: KU219940 for PmPV1-1, KU219941 for PmPV1-2, KU219942 for PmPV1-4a, and KU219943 for PmPV1-4b. The potential subunits were uploaded with accession numbers MF489085 for PmPV1-5 and MF489086 for PmPV1-6.

#### Phylogenetic and bioinformatic analysis

Multiple alignment between PmPV1 and PcOvo orthologous sequences from *P. maculata* and *P. canaliculata* shared several fully conserved sites (Supplementary Fig. 1b), and a conserved motif (GGPG) in PmPV1 subunits (Supplementary Fig. 1a).

**Figure 1:**
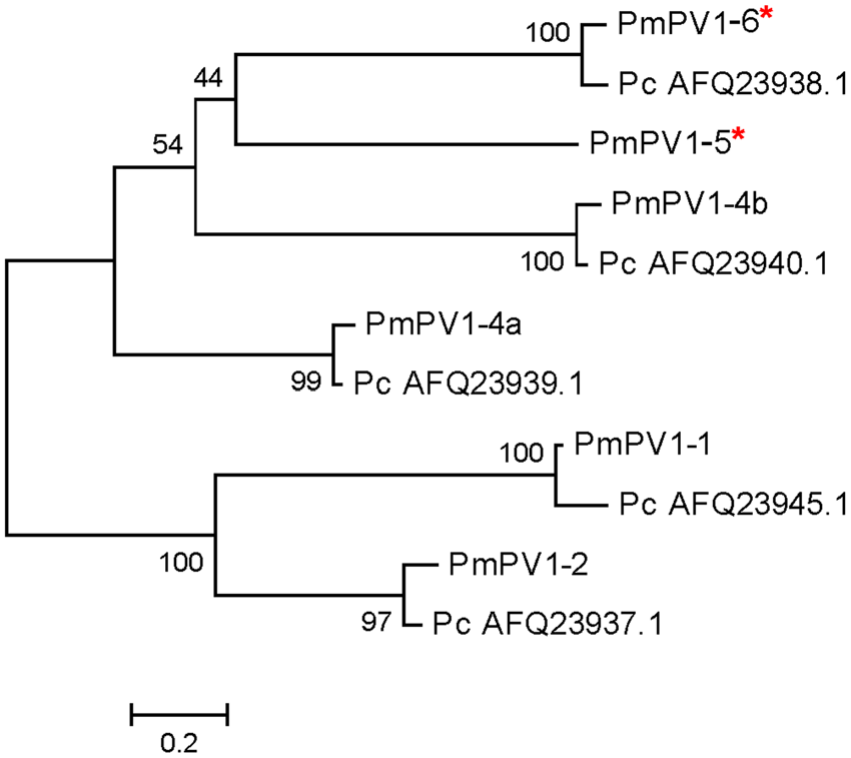
Phylogenetic tree of PmPV1 and PcOvo subunits. Phylogeny for the pairs of orthologues. Percentages in the maximum likelihood analysis in RAxML are shown near the nodes. Pc and Pm prefixes indicate *P. canaliculata* and *P. maculata*, respectively. *Indicate potential subunits.

Phylogenetic analysis indicated the presence of clades, with each pair of orthologous sequences between PmPV1 and PcOvo subunits clustering in the same clade (Fig. 1). Comparison of amino acid sequences for each pair of orthologues indicated high similarity (89.2–94.4%). However, within PmPV1, the number of similarities between any two subunit genes was significantly lower, ranging 18.4–39.4%.

#### Size and global shape of PmPV1

The size and global shape of the quaternary structure of PmPV1 were determined by SAXS. A gyration radius (Rg) of 41.80 ± 0.01 Å was estimated from the Guinier plot. This result is compatible with a compact oligomer of about 294 kDa, which is the molecular mass previously determined for PmPV1^25^. The Kratky plots of native PmPV1 are bell-shaped, as expected for globular proteins. The particle shows an anisometric shape as the pair distribution curves revealed. The maximum particle dimension (*D*_max_) is 143.0 Å and the P(r) curve exhibits a major peak at 53.2 Å. The datasets and 3D models generated are available in the SASBDB repository, https://www.sasbdb.org/data/SASDBJ7/jw90ll95gb/ with accession code: SASDBJ7.

### Structural stability of PmPV1

#### Chemical stability

Using equilibrium unfolding experiments, the overall chemical stability of PmPV1 was assayed. The spectral CM was used to estimate the population of unfolded PmPV1 (ƒU) at each GdnHCl condition. The GdnHCl unfolding transition reaches a plateau and experimental data fits a two-state model with PmPV1 completely folded between 0 and 1.5 M and completely unfolded beyond 4 M (Supplementary Fig. 2a).

#### Effect of pH and temperature

**Figure 2:**
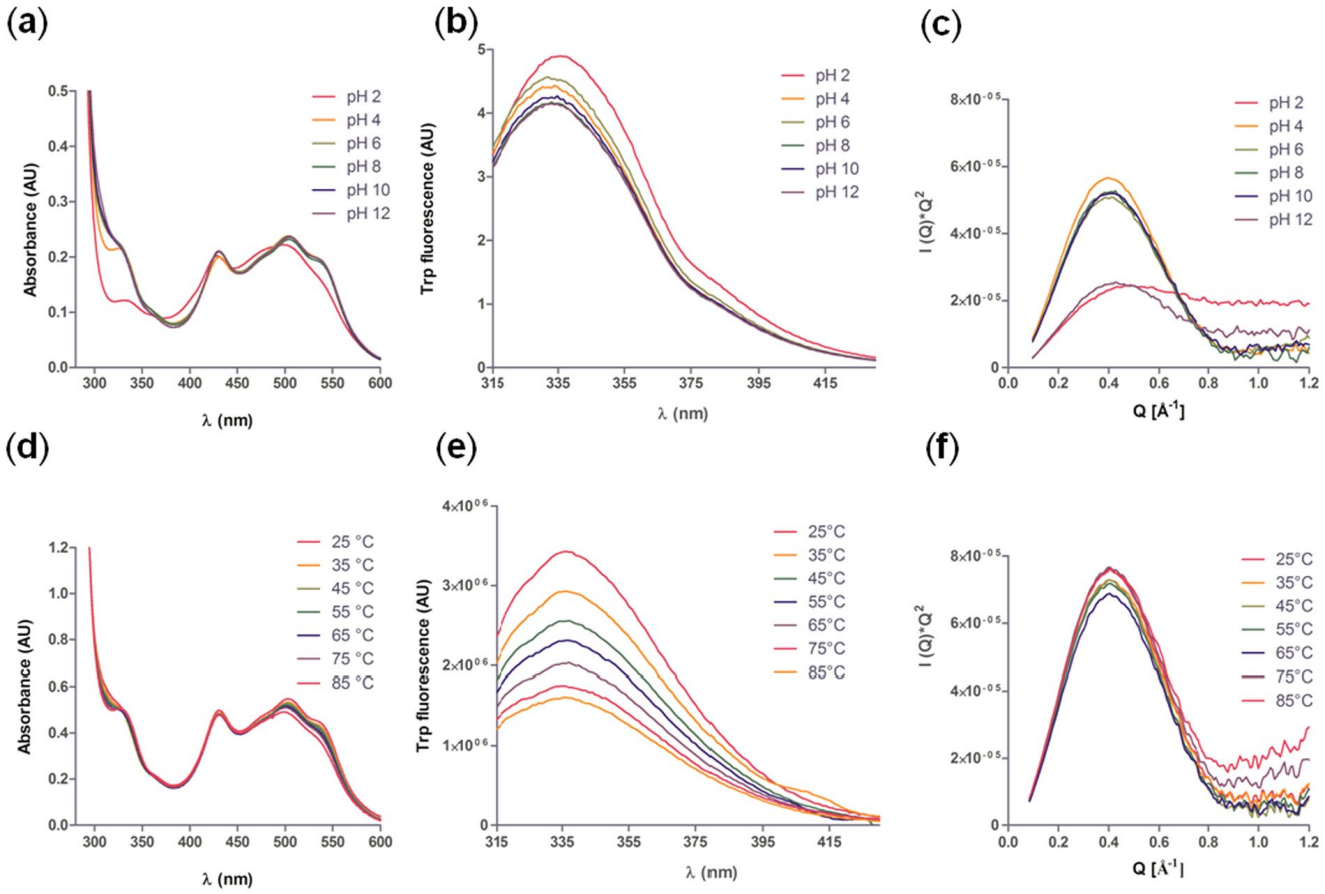
Effect of pH and temperature on the structural stability of PmPV1. (**a**) Absorption spectra in the visible region of the spectrum (25 °C). (**b**) Tryptophan fluorescence emission spectra at 25 °C. (**c**) Kratky plots obtained from SAXS data. (**d**) Absorption spectra of PmPV1 in the visible region of the spectrum at 25–85 °C (pH 7.4). (**e**) Tryptophan fluorescence emission spectra of PmPV1 at 25–85 °C. (**f**) Kratky plots obtained from SAXS data at 25–85 °C.

The structural stability of PmPV1 at different pH values showed a minor change in the fine structure of the visible region of the absorption spectrum at pH 2.0, and a slight change in the 340 nm band at pH 4.0, whereas no modification was observed in higher pH conditions (Fig. 2a). The same pattern was found in the fluorescence emission spectra, where there was a red-shift of the maximum emission only at pH 2.0, indicating the exposure of some tryptophan residues to the aqueous environment (Fig. 2b). In agreement with these results, the Rg of PmPV1 was constant between pH 2.0 and 12.0 (43.13 ± 1.71 Å). Moreover, no changes in its globularity could be detected in the Kratky plots under the different pH values, except for an evident loss of globularity at pH 2.0 and a minor loss at pH 12.0 (Fig. 2c).

There was little effect of temperature in the 25–85 °C range on PmPV1 (Fig. 2d–f **)**. There were no abrupt changes in the absorption spectra, except for a minor blue shift at increasing temperatures in the 490–550 nm region (Fig. 2d). No changes in the maximum emission were observed in the tryptophan fluorescence emission spectra between 20–70 °C (Fig. 2e). SAXS experiments showed neither a change in the Rg value (not shown) nor a loss in the globularity of PmPV1 up to 85 °C (Fig. 2f). Additionally, the unfolding effect of pH and temperature compared with that of GdnHCl, highlights the lack of structural changes exerted by the former two in the wide range of conditions assayed (Supplementary Fig. 2a **)**.

**Figure 3:**
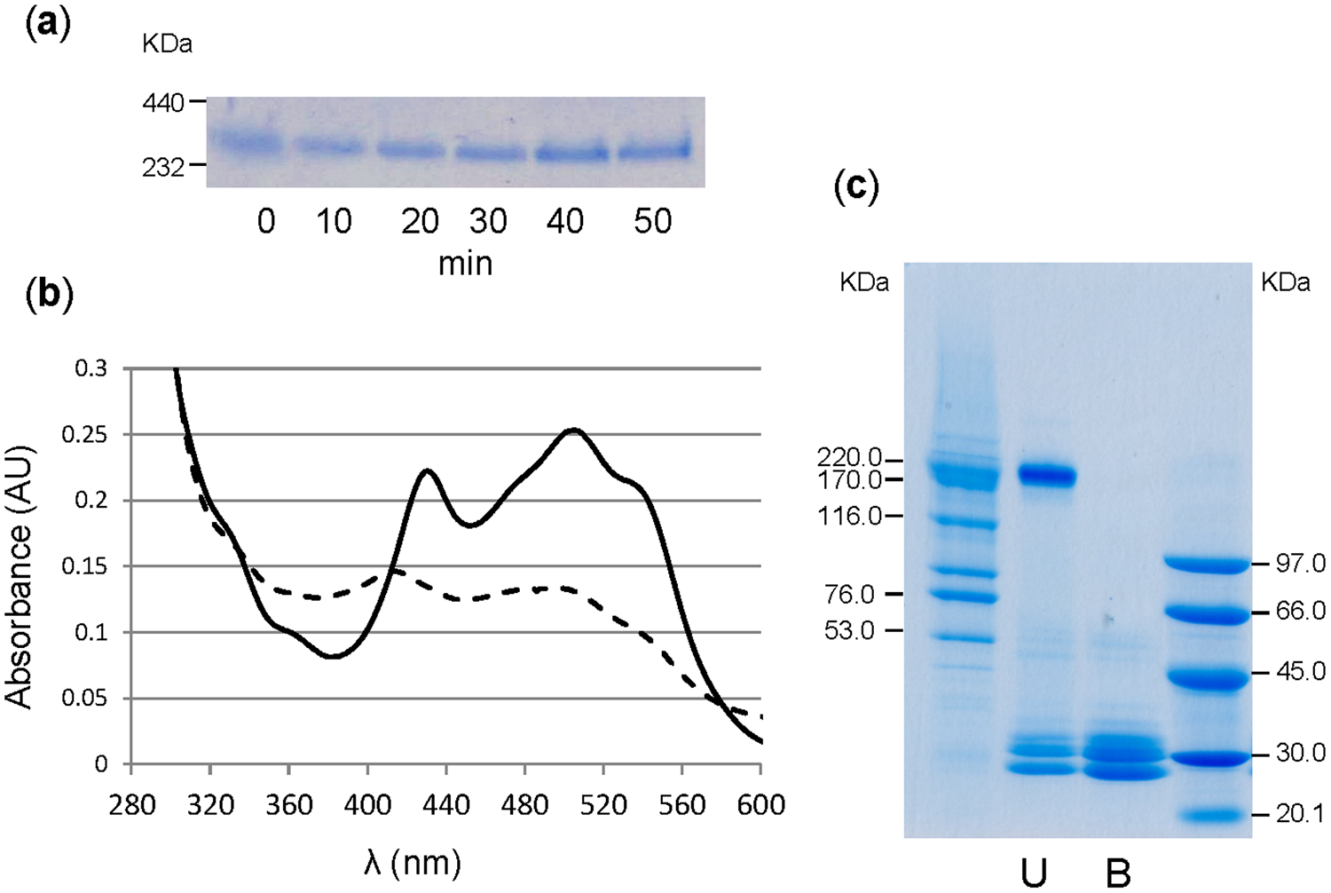
Thermal and kinetic stability of PmPV1. (**a**) Behaviour of PmPV1 in native PAGE boiled for 0–50 min. (**b**) Absorption spectrum of PmPV1 before (solid line) and after (dashed line) boiling for 50 min. (**c**) SDS-PAGE analysis of PmPV1 SDS-resistance assay. The same PmPV1 sample was unheated (U) or boiled (B) in the presence of SDS for 10 min immediately prior to be loaded into the gel.

Moreover, prolonged boiling (50 min) of PmPV1 in native (non-denaturing) conditions did not affect the electrophoretic mobility of the oligomer, though the possibility that it could have refolded and reassembled once the temperature was decreased could not be excluded (Fig. 3a, full-length gel is presented in Supplementary Fig. 2b). A decrease in the intensity of absorbance in the carotenoid region was evident, probably due to the damage of the thermolabile carotenoid pigment (Fig. 3b).

#### Resistance to sodium dodecyl sulphate

Proteins with a high energetic barrier between the folded and unfolded states are very resistant to unfolding and are described to be kinetically stable^30,31^. Comparison of the migration of unheated and boiled PmPV1 samples previously incubated with SDS showed a significantly slower migration of most of the unheated PmPV1 oligomer, indicating that it was at least partially resistant to SDS-induced denaturation (Fig. 3c). The slower migration is a sign of decreased SDS binding and consequently of a lesser overall negative charge of the SDS-protein complex compared to the fully SDS-bound (denatured) protein^30^.

#### Capacity to withstand digestion by *in silico*, *in vitro* and *in vivo* assays

*In silico* digestion analysis shows that PmPV1 is a fully digestible protein. However, the SDS-resistance, the high structural stability in a wide range of pH (pH 4.0–12.0), combined with the information from related perivitellins^7,24^ suggested that PmPV1 could withstand the gastrointestinal tract of a predator without perturbations in its structure. We tested this by *in vitro* and *in vivo* assays with physiologically-relevant digestion systems. First, we performed a digestion with pepsin and trypsin, mimicking the conditions found in the gastrointestinal tract of mammals; this allowed comparisons among species, as these assays were previously performed for other *Pomacea* perivitellins^7,24^. In another experiment, the digestion resistance to other intestinal proteases was evaluated. Also, an *in vitro* high-protease assay using pancreatin was used to mimic duodenal digestion. These *in vitro* assays were complemented by an *in vivo* experiment in mice. Finally, the activity of a non-gastrointestinal protease, fungal proteinase K, against PmPV1 was assayed *in vitro*.

An *in-silico* enzymatic digest of PmPV1 subunits and BSA was performed with gastric pepsin, duodenal released trypsin, chymotrypsin and elastase, and fungal proteinase K. The number of potential cutting sites of these proteases ranged from 65 to 100 considering all subunits indicating that it is a fully digestible protein. Normalization of PmPV1 subunits and BSA for a 20 kDa subunit highlights the similarity on the amount of cutting sites between them. The numbers of cutting sites for each enzyme of each subunit are shown (Supplementary Table 1).

PmPV1 resists cleavage when exposed sequentially to 2 h of gastric and duodenal phases (Supplementary Fig. 3a). In the same manner, we studied the effect of other duodenal proteases and a mixture of all of them. PmPV1 resisted the proteolytic activity of elastase, α-chymotrypsin alone or in a mixture containing trypsin, elastase and α-chymotripsin. The control was digested in all cases (Supplementary Fig. 3b).

PmPV1 was incubated with pancreatin, a mixture of lipase, amylase and pancreatic proteases. Digestion products, monitored by electrophoresis, showed that BSA (control) was readily degraded, whereas PmPV1 maintained its electrophoretic behaviour for up to 120 min (Supplementary Fig. 3c). Proteinase K, a fungal protease with broad specificity, was employed to perform a limited proteolysis of PmPV1. Incubation with different protease concentrations showed no evidence of PmPV1 degradation, while BSA was completely digested (Supplementary Fig. 3d).

#### *In vivo* digestibility, capacity to inhibit proteinases and haemagglutinating activity of PmPV1

**Figure 4:**
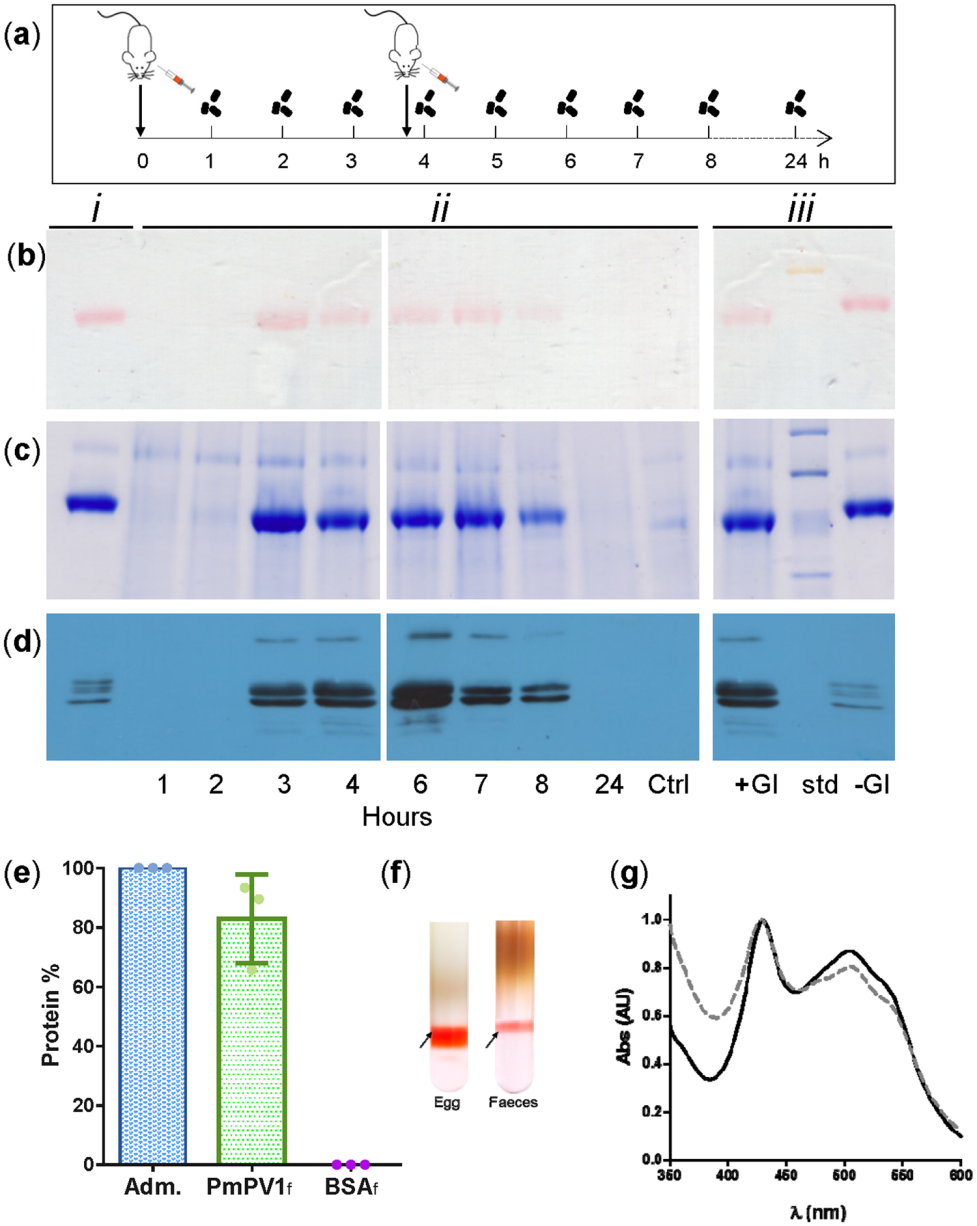
*In vivo* digestibility of PmPV1. (**a**) Experimental protocol showing oral administration of PmPV1 and faeces collection times. (**b**) Native PAGE without staining, showing coloured PmPV1; (**c**) Coomassie staining of the same gel shown on (**b**). (**d**) Western blot of faecal protein antibody known to cross-react with PmPV1 subunits. In panels (b–d): *i*. Purified PmPV1. *ii*. Faecal proteins showing PmPV1 in faeces collected 1–24 h after first administration. Ctrl: Faecal proteins collected in the control mouse 4 h after beginning the experiment. *iii*. Comparison of PmPV1 with (+GI) or without (−GI) passage through the gastrointestinal tract. Molecular weight marker (std): thyroglobulin (669 kDa), ferritin (yellowish-coloured, 440 kDa), catalase (232 kDa), lactate dehydrogenase (140 kDa). (**e**) Percentage of PmPV1 recovered in faeces (PmPV1_f_) ± 1 SD, after a single oral administration (Adm) to mice (n = 3). BSA_f_ (control) was not detectable in faeces above 0.1% (n = 3). Dots represent the individual mice data. (**f**) Isopicnic flotation of PmPV1 (arrows) after NaBr ultracentrifugation of the 100,000 × g supernatants of *Pomacea maculata* eggs (Egg) and faeces of PmPV1-fed mice (Faeces). (**g**) Absorption spectra of PmPV1 before (full line) and after (dashed line) the passage through mice gastrointestinal system.

After oral administration of PmPV1 to mice, faecal proteins were analysed. The amount of PmPV1 administered was very little (2.4 mg), equivalent to the quantity contained in 0.1 g of eggs (a single egg clutch weighs 10–20 g), and corresponded to 0.7% of mouse daily protein requirement. The presence of PmPV1 within the faeces was assessed by native PAGE and WB and quantified by ELISA. An electropherogram photograph before any stain was added to the gel clearly showed a coloured high-molecular-weight protein corresponding to the naturally-coloured PmPV1, which was observed in the faeces 2–8 h after PmPV1 administration (Fig. 4a–c, full-length gels are presented in Supplementary Fig. 4a,b). Nearly 83% of the PmPV1 administered was recovered in faeces (Fig. 4e) whereas BSA was not recovered at least above 0.1%. The electrophoretic mobility of purified PmPV1 in extraction buffer and PmPV1 from faeces was only slightly different in native PAGE, while its subunit pattern with or without passage through the digestive tract was indistinguishable (Fig. 4c, full-length blots are presented in Supplementary Fig. 4c). Moreover, the hydration density, as well as the absorption spectra maximum of PmPV1 isolated from eggs and faeces were the same **(**Fig. 4f,g).

To determine if the high resistance to proteolysis was due to a PmPV1 protease inhibition capacity, two assays were performed. When PmPV1 was preincubated with pepsin for 10 min before BSA was added, the analysis of the digestion products revealed that pepsin retained its proteolytic activity (Supplementary Fig. 5a). A trypsin inhibition test showed no change (*P* > 0.05) in the trypsin specific activity after co-incubation with PmPV1 (Supplementary Fig. 5b).

Considering reports on the presence of carotenoproteins with lectin-like activity in *Pomacea* ^24^, we tested for haemagglutinating activity against rabbit erythrocytes. No haemagglutinating activity could be detected up to 3.28 µg/µL of PmPV1 (not shown).

## Discussion

### PmPV1 is only related to other Pomacea carotenoproteins and is a kinetically stable protein

Our understanding of the structure and role of animal (egg) proteins in defence against predators lags far behind from those involved in defence against pathogens. This study provides evidence that eggs from a freshwater snail have evolved plant-like non-digestible KSP with a potential antinutritive defensive role with a strong phylogenetic relationship only with proteins from congeneric species. Sequence similarities among PmPV1 subunits were moderate (18–39%); however, *P. maculata* and *P. canaliculata* orthologues shared high sequence similarity (89–94%) suggesting a low amino acid divergence for the pairs of orthologues between the subunits of these congeneric species. Overall, these may indicate that gene duplication may have occurred before speciation, which is also in agreement with the previous analysis of PcOvo genes from *P. canaliculata*, indicating that carotenoprotein subunit duplications occurred early in the evolution of the genus *Pomacea* ^13^. The restricted distribution of these proteins to one ampullariid genus suggests a recent origin. In fact, it has been estimated that *Pomacea* evolved and diversified shortly after the separation of Africa and South America in the early Cretaceous^26^. This contrasts with other *Pomacea* egg proteins, such as protease inhibitors, that are widespread in nature.

PmPV1 has evolved a remarkable kinetic stability towards the aggressive digestive environment that co-opted to new functions, notably an antinutritive role as a non-digestible macromolecule. Kinetic stabilization is likely required when proteins work under harsh extracellular conditions in which deleterious alterations such as proteolysis, or undesirable interactions with other macromolecular components are prone to occur. This study provides evidence that *P. maculata* eggs possess a defensive strategy against predation, similar to that of plant seeds. In this regard, the digestibility of seeds also depends largely on the abundance of KSP, as a strong correlation between its non-digestibility and the abundance of KSPs in seeds were reported^6^.

Indeed, the adaptive evolution of storage protein genes into KSP non-digestible genes may have occurred in response to gastrointestinal environmental conditions. Although this has not been tested in other egg carotenoproteins of the genus, PmPV1 shares similar structural stability under extreme pH and temperatures with PcOvo and PsSC. The three oligomeric carotenoproteins can withstand a pH range that covers the environment encountered in most digestive systems^22,32^. In addition, considering that apple snails are mesophilic organisms that cannot tolerate extremes of heat or cold^33^, it is interesting to note that the three perivitellins show high stability^24,34^ at temperatures that usually denature mesophilic proteins. It seems that selective pressures have favoured temperature-insensitive mutations, causing them to maintain proper folding and stability. To further understand the remarkable structural stability of these macromolecules toward these varied agents, it is important to consider their heavy glycosylation (13–18% by wt)^25,35^. It is generally believed that glycosylation can significantly influence thermal stability, resistance to degradation and quaternary structure of a protein^36^. PmPV1 oligomer and subunit sizes suggests that it may be 12–14-mer that does not have disulphide bonds among subunits (Supplementary Fig. 6). Besides hydrophobic interactions do not play a significant role in stability at high temperatures. It is therefore possible that glycosylation may be involved in providing the unusually high structural stability when the particle is exposed to perturbations by pH, temperature or chaotropic agents. Another indication of PmPV1 stability was its resistance to pepsinolysis, as pepsin requires a certain degree of flexibility in its substrates (a 5–8 residue section of a substrate protein must lie across its active site in an extended conformation)^37^. Furthermore, the *in silico* analysis showed that the average number of cleavage sites for digestive proteases in PmPV1 did not differ from that of a fully digestible protein, suggesting these sites were not under selective pressure. Protein resistance to SDS and proteolytic treatments are strongly correlated with their structural rigidity^38^. In contrast to thermal denaturation, SDS is thought to unfold proteins largely by competing for hydrogen bonds with the polar groups of the backbone and side chains. Structural basis of PmPV1 kinetic stability is indicated by both its migration pattern in SDS-PAGE and the oligomer resistance to proteolytic treatment, a characteristic shared by most KSPs^30^. At present, the knowledge on invertebrate perivitellin 3D structures is limited to *P. canaliculata* major perivitellins, PcOvo^22^ and PcPV2^17^. The low-resolution model of PmPV1 obtained in this study is similar in size and global shape to that of PcOvo.

### PmPV1 is a defensive macromolecule with potential antinutritive properties

Peptide and amino acid uptake is of central significance in the protein nutrition of animals^39^. Ingested amino acids are released in the small intestine of predators by peptidases^39^. In addition, the colon also contains substantial amounts of peptidases largely provided by the intestinal flora^40^ that use the undigested proteins or peptides as substrate^41^. In this regard, egg storage proteins, while providing a rich source of amino acids for the developing embryo^42,43^, offer a high nutritional source to a predator or pathogen, a major reason eggs are often subjected to intense predation^44,45^. However, in addition to protein quantity, evidence indicates that dietary protein quality also has a significant impact on predator-prey interactions. The factors limiting the nutritional value (bioavailability) of proteins can include either predator factors such as digestive capacities, or prey factors such as proteases inhibitors or KSPs. In this regard, the storage carotenoproteins from *Pomacea* eggs, while a major source of nutrients during embryo development^7,15,42^, are not bioavailable for predators, as their high stability renders them a non-digestible protein. In particular, we provide evidence that the perivitellin PmPV1, which has no proteinase inhibitor activity, is extremely resistant to several proteases *in vitro*, even at high, non-physiological concentrations, indicating it is an intrinsically non-digestible protein. Its poor nutritional value was also tested *in vivo* by oral administration of PmPV1 to mice. We found that it resists the harsh gastrointestinal passage, whereas BSA was fully digested as previously reported. Furthermore, PmPV1 protein integrity was maintained after passing through the gastrointestinal tract and excretion in the faeces. This was reflected in the lack of alteration in the non-covalent binding between the carotenoid and the protein by the harsh environment encountered in the gut of rodents, known predators of apple snails^12^. This indicates that this perivitellin is neither digested in the small intestine nor fermented by the microorganisms in the large intestine. These results, together with the fact that PmPV1 is massively accumulated in eggs (up to 64% of the egg protein^28^) indicate that *P. maculata* eggs are a poor source of proteins and amino acids for most predators, because of its low digestibility which renders it an antinutritive protein. To our knowledge, this is the first evidence in animals of the association between kinetic stability of a storage protein and poor nutritional value.

PmPV1 also has no haemagglutinating activity, which is similar to PcOvo but contrasts with the orthologue PsSC, a potent lectin. The acquisition of these different functions in a relatively short timeframe would also be explained by their high stability, as it has been shown that protein’s capacity to evolve is enhanced by the mutational robustness conferred by extra stability^46^. Protein stability promotes evolvability by allowing a greater number of mutational (and structural) changes while still folding in its native structure. The retention of the basic native structure in turn, is normally a prerequisite for the acquisition of new functions. In this regard this work provides evidence highlighting that these hyperstable storage proteins are emerging as a family of multifunctional carotenoproteins exclusive to *Pomacea* snails.

## Conclusions

All organisms face an unceasing onslaught from potential predators and pathogens. Not only have a variety of defence mechanisms evolved, but they have done so repeatedly. The same challenge is met by much the same solution. One line of protection is to lower the nutritious value of compounds, a characteristic found in several plant seeds. Our study provides experimental evidence that the selective pressure of the harsh gastrointestinal tract conditions found in potential predators on PmPV1 has resulted in the acquisition of evolutionary novelty; this modulated the conformational stability and structural rigidity of the most abundant perivitellin in order to enhance its resistance to degradation. Its structural and functional properties are only similar to the antipathogen and antipredator parts of the protection system of plant seeds. This is, to our knowledge, the first report of a kinetically stable storage protein that is non-digestible and hence lowers the nutritional value of the prey (egg) further extending the presence of plant-like antinutritive KSPs in animals, albeit achieved with unrelated proteins representing an intriguing form of convergent evolution between plant and animal embryo defences. The paramount importance of PmPV1 and related carotenoproteins in *Pomacea* egg colouration, and the critical role they play in survival and development suggest that the early origin of their subunit sequences (gene family) significantly contributed to the success of this group of molluscs. This supports the hypothesis that the unique features of these storage proteins may be associated with aerial egg-laying ampullariids^15,47^.

PmPV1 antinutritive features provide a description of an animal defence model where no trade-off is needed between embryo nutrition and embryo defence because both the reserve and the secondary defence are encoded in the same molecule. Since *P. canaliculata* and *P. maculata* are very closely related and are, in opposition to other congeners, very successful invasive species, the presence of related non-digestible storage proteins may indicate evolutionary novelty that may have conferred them a survival advantage. Proteins involved in reproduction appear to be evolving rapidly in these snail taxa, but further work on more distantly related species is needed, especially for those members of the family that lay eggs under water, in order to further understand the origin and evolution of these defensive proteins.

## Methods

### Sample collection

Adult *P. maculata* were collected in the Paraná River off San Pedro, Argentina (33°39′35.97″S, 59°41′52.86″W), and kept in the laboratory (voucher specimens were deposited in the Museo de La Plata Collection (MLP 13749)). Eggs were collected within 24 h of deposition by the female, and kept at −20 °C until processed.

### Purification of PmPV1

This protein was purified from newly laid *P. maculata* egg clutches as previously described^25^. Total protein was quantified following the method described by Lowry *et al*.^48^ using bovine serum albumin (BSA, Sigma cat. 7906) as standard. Purity was checked by polyacrylamide gel electrophoresis.

### Subunit sequences and alignment

Fresh albumen gland tissues from three females were used for RNA extraction. RNA quality was checked by agarose gel electrophoresis and Agilent 2100 Bioanalyzer (Agilent Technologies, Santa Clara, CA, USA). High quality RNA sample were used for transcriptome sequencing. Transcripts were assembled by Trinity^49^ and the obtained transcriptome data of *P. maculata* ^50^ were translated into amino acid sequences. N-terminal sequences of PmPV1 subunits^25^ were used to pick up the corresponding protein and DNA sequences. The presence of PmPV1 subunits were further identified by mass spectrometric data obtained by proteomic analysis of *P. maculata* perivitelline fluid^51^. Other potential subunits were identified by homology searching *P. maculata* albumen gland transcriptome. Sequence alignment was performed using Clustal Omega (1.2.1)^52^. Signal peptides of each subunit were predicted by SignalP 4.0 server^53^ and confirmed by N-terminal sequences of PmPV1 subunits obtained by Edman degradation^25^. The expected subcellular pathways were determined using TargetP^54^. Theoretical molecular masses were calculated with the Expasy ProtParam tool^55^. Potential phosphorylated sites were predicted with DISPHOS 1.3 with default settings^56^ while N-glycosylation sites were predicted with NetNGlyc 1.0 server.

### Orthologous and phylogenetic analysis

A local BLAST was applied to search the orthologous sequences between *P. maculata* and *P. canaliculata* egg carotenoprotein subunits. Sequence alignment was performed using MUSCLE implemented in MEGA 6 with default settings. In order to determine the divergence of several pairs of orthologues in the two species of snails, PmPV1 and potential PmPV1 subunits and PcOvo subunit sequences were subjected to phylogenetic analysis using MEGA.6. The maximum likelihood method and 1000 bootstrap replicates were applied to construct the tree. Default settings were applied for other options.

### Global shape

Small angle X-ray scattering (SAXS) experiments were performed at the D02A-SAXS2 (Laboratório Nacional de Luz Síncrotron, Campinas, Brazil) as previously reported^22^. The experiments were performed at 25 °C (except for the thermal stability assay). At least three independent curves were averaged for each single experiment and analysed using the ATSAS package 2.6.0^57^.

The low-resolution model of PmPV1 was obtained from the algorithm built with DAMMIN and DAMMIF programs^58^. The resulting dummy atom model represents the shape of the scattering particle. The average of the best ten models fitting the experimental data was obtained with DAMMIF. Data were deposited in the Small Angle Scattering Biological Data Bank SASBDB.

### Chemical denaturation

The intrinsic fluorescence emission of PmPV1 tryphtophans was used to follow the PmPV1 denaturation induced by guanidine hydrochloride (GdnHCl) (Sigma). Chemical denaturation was performed by incubating overnight 50 µg/mL PmPV1 in the presence of increasing concentrations (0–6 M) of GdnHCl buffered with 0.1 M phosphate buffer at pH 7.4 at 8 °C.

Protein intrinsic fluorescence spectra were recorded on an Olis-upgraded SLM4800 spectrofluorometer (Olis Inc., Bogart, GA) coupled with a Lauda Alpha RA 8 thermostatic bath. Fluorescence spectra were recorded in emission scanning mode at 25 °C. Tryptophan emission was excited at 295 nm (8 nm slit) and recorded between 315 and 436 nm (8 nm slit). Three spectra were recorded and averaged for each sample. The corresponding buffer blank was subtracted. At least two independent samples were measured.

Spectra were characterized by their center of mass (CM) calculated using equation (1).

1 $${\rm{CM}}=\frac{{\sum }^{}{\rm{I}}({\rm{\lambda }})\cdot {\rm{\lambda }}}{{\sum }^{}{\rm{I}}({\rm{\lambda }})}$$

Where “I” is fluorescence intensity and “λ” represents the wavelength.

The populations associated with the unfolded fraction (ƒu) were calculated from the CM using equation (2).

2 $${f}{\rm{u}}=\frac{{{\rm{CM}}}_{0{\rm{M}}}-{{\rm{CM}}}_{{\rm{xM}}}\,}{{{\rm{CM}}}_{0{\rm{M}}}-{{\rm{CM}}}_{6{\rm{M}}}}$$

Where “x” represents GdnHCl molarity in each condition.

### Effect of pH and temperature on structural stability

To study the effect of pH on PmPV1 structural stability, the protein was incubated overnight in different buffers ranging from pH 2.0 to 12.0 following a previously reported method^24^. Samples were analysed by absorbance and fluorescence spectroscopy and by SAXS. Absorbance spectra of were recorded between 300–650 nm in an Agilent 8453 UV/Vis diode array spectrophotometer (Agilent Technologies, Waldbronn, Germany) taking advantage of the fact that PmPV1 is a carotenoprotein and the protein-carotenoid interaction could be followed by its spectrum in this range. Fluorescence emission was recorded as described in the *Chemical denaturation* section. Two independent samples were measured three times and the corresponding buffer blank was subtracted. The effect of temperature on PmPV1 at pH 7.4 was also measured by absorption and fluorescence spectroscopy and by SAXS in the range 25–85 °C. The effect of extreme thermal conditions was analysed by boiling PmPV1 for 50 min and evaluating the oligomer integrity using native (non-denaturing) gel electrophoresis and the carotenoprotein fine spectra using absorbance spectrophotometry.

### Resistance to sodium dodecyl sulphate

Resistance to sodium dodecyl sulphate (SDS)-induced denaturation serves to identify proteins whose native conformations are kinetically trapped in a specific conformation because of an unusually high-unfolding barrier that results in very slow unfolding rates^36^. The resistance to SDS was assayed following the Manning and Colon^30^ procedure. Briefly PmPV1 in Laemmli sample buffer (pH 6.8) containing 1% SDS was either boiled for 10 min or unheated prior to its analysis by 4–20% SDS-PAGE. The gels were then stained with Coomassie blue.

### *In silico* digestion of PmPV1

To determine PmPV1’s susceptibility to digestion by the gastrointestinal tract enzymes and proteinase K, an *in silico* digestion was performed using on-line software ExPaSy PeptideMass^59^ and MS-Digest (http://prospector.ucsf.edu), comparing the data with BSA. The number of cutting sites was normalized to a 20 kDa subunit.

### *In vitro* gastrointestinal digestion

A simulated gastroduodenal digestion of PmPV1 was performed under gastric followed by duodenal conditions using the method described by Moreno *et al*.^60^ with some modifications, as follows. PmPV1 in double-distilled water was dissolved in simulated gastric fluid (SGF) (0.15 M NaCl, pH 2.5) to a final concentration of 0.5 µg/µL. Digestion commenced by adding porcine pepsin (Sigma, cat. P6887) at an enzyme:substrate ratio of 1:20 (w/w). Gastric digestion was conducted at 37 °C with shaking for 120 min. Aliquots of 5 µg protein were taken at 0, 60 and 120 min for SDS-PAGE. The reaction was stopped by increasing the pH with 150 mM Tris/Cl buffer pH 8.5 and samples were immediately boiled for 5 min in SDS electrophoresis buffer with β-mercaptoethanol (4%) and analysed as described above.

For *in vitro* duodenal digestion, 100 µL of the 120 min gastric digest was used as starting material. The pH of the digests was adjusted to 8.5 with 0.1 M NaOH and the following were added: 22.8 µL 0.15 M Tris/HCl (pH 8.5) and 4.17 µL 0.25 M sodium taurocholate (Sigma). The simulated duodenal digestion was conducted at 37 °C with shaking using bovine pancreas trypsin (Sigma cat. T9935, 1.92 µg/µL in HCl 1 mM) at an enzyme:substrate ratio of 1:2.8 (w/w). Aliquots were taken at 0, 60 and 120 min for SDS-PAGE analysis. BSA was used as positive (with enzyme) and negative (without enzyme) control in both gastric and duodenal digestion.

Digestions with elastase, α-chymotrypsin and a mixture of the three duodenal proteases were performed following a similar procedure as with trypsin, starting with PmPV1 in SFG and the following enzymes were added: elastase (Sigma cat. E0258, 0.5 µg/µL in HCl 1 mM), using an enzyme:substrate ratio of 1:10 w/w, and α-chymotrypsin (Sigma, cat. C3142, 1.92 mg/mL in 1 mM HCl), at an enzyme:substrate ratio of 1:2.8 w/w. Then, a mixture of the three proteases (Trypsin/α-Chimotrypsin/Elastase) was assayed in an enzyme:substrate ratio of 1:1:0.28:2.8 (by wt).

### High-protease assay: pancreatin and proteinase K treatment

The high protease assay described by Mandalari et·al.^37^ was performed. Briefly, 30 µL of 2.5 µg/µL BSA as control protein or 30 µL of PmPV1 (2.7 µg/µL in distilled water) were added to vials containing standard intestinal fluid (150 µL), which consisted of pancreatin (10 mg/mL, Sigma, P7545) in 67 mM K_2_HPO_4_ pH 7.6 with a final pancreatin:substrate ratio of 1:9.6. In parallel, standard intestinal fluid and PmPV1 (without pancreatin) were run as controls. Samples were incubated in a water bath at 37 °C and aliquots were taken at intervals of 20 min. Aliquots were analysed by SDS-PAGE as described above.

Proteinase K treatment was performed following Kim *et al*.^61^ using the concentrations modified by Frassa *et al*.^17^. PmPV1 (1 mg/mL) was incubated with proteinase K (1, 10 and 100 µg/mL) in 50 mM Tris/HCl buffer (pH 8.0) containing 10 mM CaCl_2_ at 37 °C for 30 min. Digestion was ended by boiling samples in SDS sample buffer, and products were analysed by SDS-PAGE as above.

### *In vivo* digestibility of PmPV1

The experiment performed with mice was approved by the “Comité Institucional para el Cuidado y Uso de Animales de Laboratorio” (CICUAL) of the School of Medicine, Universidad Nacional de La Plata (UNLP) (Assurance No. P 01012016) and were carried out in accordance with the Guide for the Care and Use of Laboratory Animals^62^. Female BALB/c mice used in the experiments were obtained from the Experimental Animals Lab, School of Veterinary Science, National University of La Plata (UNLP), from a colony started with a stock provided by NIH USA and bred in a specific pathogen-free environment. Animals that were 7.5 weeks old, weighing approximately 18 g at the start of the experiment, were kept in a general animal room and fed *ad libitum* with a commercial diet for three weeks before and during the assay. Three females were gavaged with 1.2 mg of purified PmPV1 twice, at 0 and 3.5 h after the first administration. Faeces from each animal were collected hourly for the first 8 h, and then 24 h after, and immediately frozen. Before faecal protein analysis, faecal extracts were prepared following Pasquevich *et al*.^63^ with some modifications. Briefly, faeces were weighed and approximately 0.12 g was resuspended in 500 µL of EDTA 30 mM pH 8.4 in PBS with a protease inhibitor cocktail 1:100 (SIGMA) and allowed to hydrate for 10 min. Then, the sample was homogenized in a Teflon homogenizer and centrifuged at 13,000 × g at 4 °C for 10 min. The pellet was discarded and total protein in the supernatant was determined^48^. The presence of undigested PmPV1 in mouse faeces was determined by separating faecal proteins in a native gel electrophoresis. Purified PmPV1 (22 µg) was used as control. Taking advantage of PmPV1’s natural pink-reddish colour, its presence was recorded photographically before gels were stained with Coomassie Blue. Further confirmation was performed by Western blot analysis visualizing PmPV1 subunits using anti-sera against PcOvo, following previously reported methodology^25^, loading 80 µg of faecal proteins in the gel. Crossreactivity between Anti-PcOvo and PmPV1 was previously assayed^25^. Purified PmPV1 (0.5 µg) was used as control. To determine the hydration density and absorption spectra of PmPV1 in faeces, PmPV1 was isolated from faeces as described above for egg PmPV1. The 100,000 × g supernatant was subjected to NaBr gradient ultracentrifugation, and the hydration density and absorption spectra of the reddish PmPV1-fraction was compared with the PmPV1 fraction from egg supernatant ultracentrifuged in parallel at 100,000 × g.

### Quantification of PmPV1 in mice faeces

Mice were orally administered with 2.1 mg of PmPV1 (N = 3) or BSA (N = 3) and faeces were collected 2–9 h after administration. PmPV1 in faeces was quantified by a competitive ELISA based on a rabbit anti-PcOvo polyclonal serum and purified PmPV1 as standard. Polystyrene plates (Immuno plate with MaxiSorp surface, Thermo Fisher Scientific) were coated with 0.5 µg/well of purified PmPV1 in PBS. After 90 min of incubation at 37 °C plates were washed with PBS and blocked with 300 µl/well of 3% skim milk in PBS overnight at 4 °C. Rabbit anti-PcOvo serum (final concentration 1:10,000 in PBS-T containing 1.5% skim milk) with serial dilutions of PmPV1 (standard curve) or the samples (faecal extracts) in PBS-EDTA were pre-incubated at room temperature for 1 h and afterwards added to the blocked plates (50 µl/well) and incubated 1 h at room temperature. Each condition was carried out in triplicate. Goat anti-rabbit horseradish peroxidase conjugate (Bio-Rad) was added (50 µl/well) at 1:3000 dilution. After 1 h of incubation at room temperature, plates were washed with PBS-T, and 50 µl/well of substrate solution (550 µM of 2-2′-azino-di-(3-ethylbenzthiazoline sulfonic acid and 0.01% H_2_O_2_) in substrate buffer (100 mM citrate/200 mM disodium hydrogen phosphate at pH 5.3) were added. Absorbance was measured at 405 nm in a microplate reader (Beckman Coulter, DTX 880). The absorbance values were transformed using logit *p* as described elsewhere^64^ where *p* is defined as equation (3)

3 $$p=\frac{({\rm{A}}-{{\rm{A}}}_{0})}{({{\rm{A}}}_{{\rm{\max }}}-{{\rm{A}}}_{0})}$$

Where A is the measured absorbance for a standard concentration or a sample, A_max_ is the absorbance measured when any soluble PmPV1 was pre-incubated with the rabbit serum and A_0_ is the absorbance when any rabbit serum were pre-incubated with sample. This transformation leaded to a lineal relation with log_10_C (C = Concentration of PmPV1).

### Western blot analysis of BSA in faeces

The presence of BSA in faeces were analysed by Western Blot, using commercial BSA as standard (SIGMA) as described above, except PBS Tween 0.1% was employed instead of skim milk. Polyclonal antibody anti-BSA (Invitrogen) (1:2,500) and goat anti-rabbit horseradish peroxidase conjugate (1:3,000) were employed.

### Pepsin and trypsin inhibition assay

To test for pepsin inhibition capacity, PmPV1 (final concentration 0.5 µg/µL) in SGF (0.15 M NaCl, pH 2.5) was incubated with pepsin (enzyme:substrate ratio 1:20 w/w) for 10 min at 37 °C. BSA (final concentration 0.5 µg/µL) was added to the vial, and positive and negative controls of BSA and PmPV1 were assayed in parallel. After 120 min, aliquots were taken and analysed by SDS-PAGE.

To test for trypsin inhibition capacity, 60 µg of PmPV1 was incubated with 4 µg of trypsin for 5 min. Trypsin activity was determined following the method of Schwert and Takenaka^65^ measuring the hydrolysis of N-benzoyl-L-arginine ethyl ester (BAEE) as an increase in absorbance at 253 nm at 25 °C. Inhibition of trypsin was evidenced as a decrease in BAEE hydrolysis. Results were expressed as trypsin specific activity (U/mg of trypsin, where U represents the amount of enzyme that causes an absorbance increase of 0.003/min at 25 °C). Three independent PmPV1 samples were measured. A Mann-Whitney test was performed to compare trypsin activity with and without PmPV1 using GraphPad Prism version 5.03 (GraphPad Software, San Diego California USA). A P value of 0.05 was taken as the level of significance.

### Agglutinating capacity of PmPV1

Rabbit erythrocytes were obtained from the animal facilities at University of La Plata (UNLP) as described elsewhere^8^. Haemagglutinating activity was assayed in microtitre U plates (Greiner Bio One, Germany) by incubating a two-fold serial dilution of PmPV1 (3.28 ± 0.15 µg/µL, n = 3) with 1% erythrocyte suspension in phosphate buffer at 37 °C for 2 h which allows to detect haemagglutinating activity with the naked eye.

### Data availability

The datasets generated during the SAXS experiments are available in the SASBDB repository (https://www.sasbdb.org/), with accession code SASDBJ7. The cDNA sequences generated were deposited in GenBank with accession numbers: KU219940, KU219941, KU219942, KU219943, MF489085 and MF489086. The datasets of MS proteomic analysis are part of Dr. Mu PhD. thesis and of a related manuscript^50^ and are available from the corresponding author on request. All other data generated or analysed during this study are included in this published article and its supplementary information files.

### Ethics Approval And Consent To Participate

The experiment with mice was approved by the “Comité Institucional para el Cuidado y Uso de Animales de Laboratorio” (CICUAL) of the School of Medicine, Universidad Nacional de La Plata (UNLP) (Assurance No. P 01012016) and were carried out in accordance with the Guide for the Care and Use of Laboratory Animals (Guide for care and use of laboratory animals. Washington: Academic Press; 1996).

## Electronic supplementary material

Supplementary Figures and a Table
