# Supplementary material for: Convergent evolution of plant and animal embryo defences by hyperstable non-digestible storage proteins
Source: Sci Rep. 2017 Nov 20;7:15848. doi: 10.1038/s41598-017-16185-9 (PMC5696525; doi:10.1038/s41598-017-16185-9)

Supplementary information

Convergent evolution of plant and animal embryo defences by hyperstable non-digestible storage proteins

María Yanina Pasquevich, Marcos Sebastián Dreon, Jian-Wen Qiu, Huawei Mu and Horacio Heras

Supplementary Table 1

Supplementary Table 1 | *In silico* digestion of PmPV1 subunits and BSA by the enzymes assayed in the *in vitro* experiments. Values represent the number of possible cutting sites in the subunits. No missing cutting sites were allowed. Values between brackets are the number of cutting sites normalized for a 20 kDa subunit to highlight the similarity among them and the control protein.

	Mw (KDa)[a]	Pepsin	Trypsin	Elastase[b]	Chymotrypsin	Proteinase K
PmPV1-1*	23.184	70 (60)	26 (22)	86 (74)	16 (14)	80 (69)
PmPV1-2	22.399	74 (66)	22 (20)	100 (89)	12 (11)	81 (72)
PmPV1-4a	21.901	69 (63)	23 (21)	92 (84)	16 (15)	76 (69)
PmPV1-4b	22.269	65 (58)	17 (15)	92 (83)	18 (16)	78 (70)
BSA	69.324	238 (69)	82 (24)	230 (66)	53 (15)	211 (61)

[a]Calculated using ExPaSy PeptideMass (Wilkins *et al.* 1997). [b]Calculated using MS-Digest (http://prospector.ucsf.edu) *Subunits named according to Pasquevich *et al.* 2014

Supplementary Results

Supplementary Figure 1 | Sequences of PmPV1 subunits and PmPV1 and PcOvo sequence alignment. (**a**) Alignment of deduced aminoacid sequences of PmPV1 subunits. A conserved sequence is marked in red. The putative signal sequences are in italics. Potential phosphorilation sites are underlined, and potential N-glycosylation sites (NXS/T) are underlined and in bold. N-Glycosylation sites (NXS/T) with low probability of being glycosylated are not shown. *: fully conserved site. (**b**) PmPV1 and PcOvo sequences alignment. Alignment was done using MEGA 6. Pc and Pm prefixes indicate *P. canaliculata* and *P. maculata*, respectively. Sequence AFQ23939.1 has been trimmed at amino acid 217. *indicate potential subunits.

(a)

```
PmPV1-1     -----MA-ATLVLLLAMSTLLTSGVEMIKHDLVFLEVDS—GSCKENASKIFQDKSMIPG
PmPV1-2     -----MT-AILALLLALSILLPSGAVEDVQDLVFAEWDK--GSSHEHACSALRNSSVIEK
PmPV1-6*    MAEHRIPVLLLLVVVAASSLAQQISPQK--HYIIYEVRNI-EKTPEEVREEMK---DTDI
PmPV1-5*    -----ML-VATLTLVALSAVFTNAYGSD--QYTILDVYRASNMSVEDYKDLLK---DLDV
PmPV1-4a    -----MY-ALAIALLAFSTFVSNTIANK--EYLLLDIRDA---TTSEIISALR---DVEI
PmPV1-4b    -----MF-VATFLLLTIATVV-VKASPH--NYLIMDIEPPKSVSESDILNLLS---PLQV
                 :        ::: : .          .  : :          ..   . :

PmPV1-1     EVSGQVIGTSKFFAVLRE-----QDLARMNLKDVKEVVPVTKLEDYLESWNASWRIH-RD
PmPV1-2     GLTVKEVGTSKFAAVLSE-----PILARLKFHGLIEAVPVEEVDIVMKRLNVSTPPA-QE
PmPV1-6*    LYSFKVLAAPSYHIVVEVNPRNMRKLEEVELKGKIRMVPVVSMMDVAESLGVSWPSSGAR
PmPV1-5*    VHSFKVLGSSRVLFVVKMREDSYEKLSKINLPGEVFSLPAGDLSDKMQDLGVEWKRW-DD
PmPV1-4a    ELKVKAKGISRHLVVVKQNDANLQKLGEIDIPGKSCSTPVEDLDNLMEDIGISWPRN--Q
PmPV1-4b    KHSFRITGSTRLLIVIRLDAQSYDKLDEITVPGKVEAIPAVNMADTMERYGVSWPRV--E
              . :  .       *:          * .: .  .     *. .:     :  . .

PmPV1-1     INETDLWLFKVSPNPKSKTLQQYDNQVRHGVRIAAPVMENFPNYILLSKGVFPPKYYIFI
PmPV1-2     ISNNNLTLIKMTPKLKGQTLQQTDAELRRLGEYMNTALSKCSHRIYISKGTFPPKIYVFL
PmPV1-6*    LPDVNLTLIERTINQEGLTTQESEAHLKGFMEELKDRLQQYNYQAFFSNGSSPPKMYIYI
PmPV1-5*    LPDANLTLFERTLELKGEPLEGLASHLKAFGEKISHVVELYPNKGFFLLGRTPPKAYVVV
PmPV1-4a    LTSVNVTLFERTLDLKDKTMEQYWSETKAYGQLVKPLLSSFTYRAFKANGAYPPKVYFFV
PmPV1-4b    LTDDNVTLFESESTLTEVTKEQLKAMLIGYGEHMSGLLQAHRFEYYQAAGATPHRHFVFV
            : . :: *::           :          .      :.          *   * : :. :

PmPV1-1     NKKEEFPK--DF-RNILDIFGGPGAVKVESLYVRILISWQ
PmPV1-2     NLPLDEIR--QF-YPALDIFGGPGSVKNEISFVQLLILRN
PmPV1-6*    NIPYEEVN--NFALIGINQFGGPAAVNTTVSFISSFPQ--
PmPV1-5*    SLPFRCRQVRYGSDFALNYLNGPGDSSTKVEFVA------
PmPV1-4a    NLPRENLN--DASSKGIDIFGGPGKARTTVQYVT------
PmPV1-4b    NSVPDEID--VFGREGVDIWGGPGDFVVKPKYVTRI----
            .                ::  .**.        ::
```

(b)

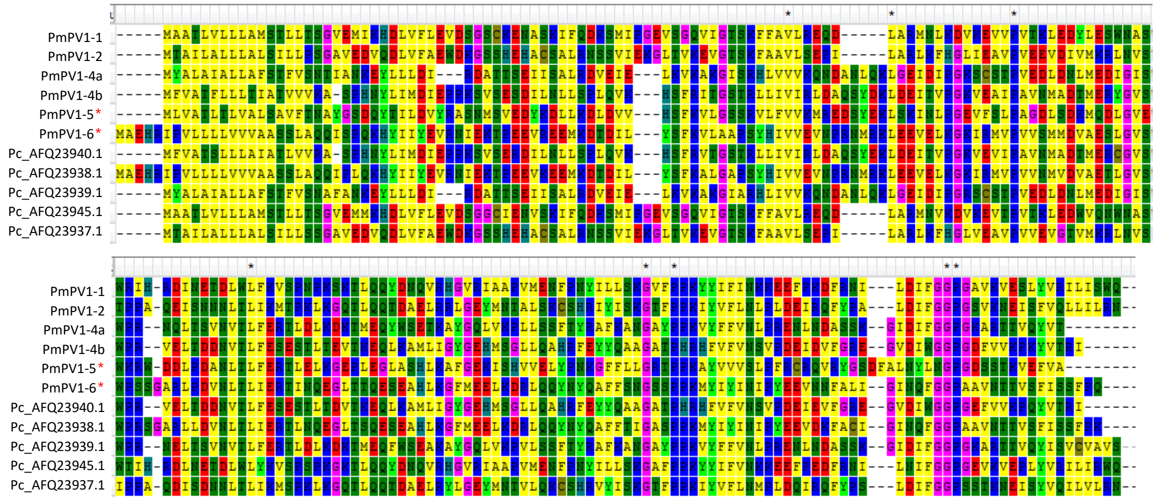

Supplementary Results

Supplementary Figure 2 | Structural stability. (a) Comparison of different denaturant agents on PmPV1 structural stability by fluorescence emission spectroscopy. Black circles: unfolding of PmPV1 population as a function of GdnHCl at pH 7.4 and 25°C; red squares: effect of pH at 25°C; green triangles: effect of temperature at pH 7.4. (**b**) Behavior of PmPV1 in native PAGE boiled for 0–50 min.

(a)

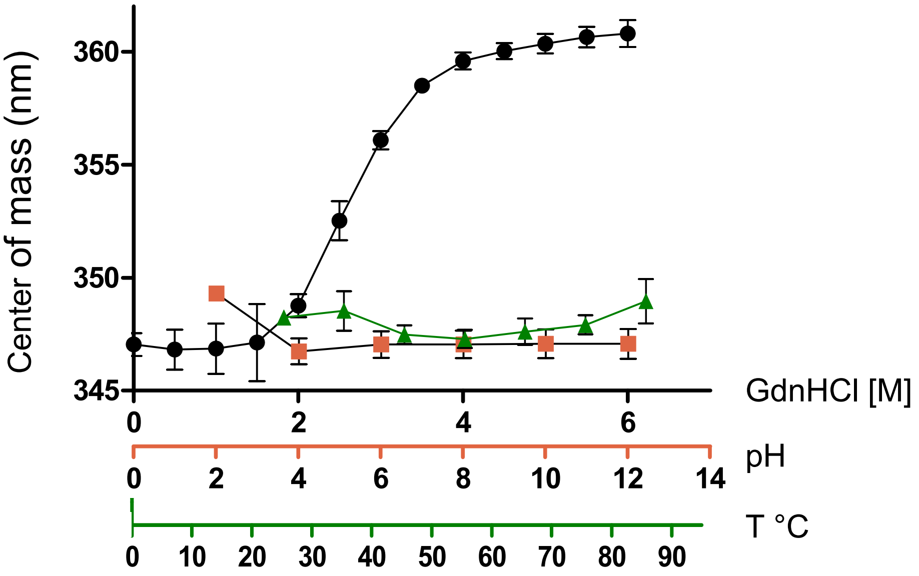

(b)

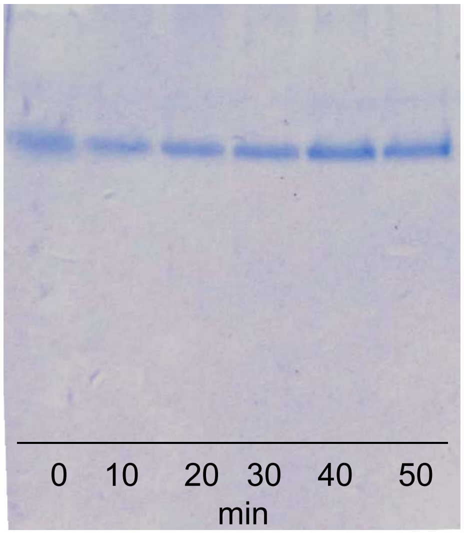

Supplementary Results

Supplementary Figure 3 | Simulated digestion of PmPV1 analyzed by SDS-PAGE. (**a**) Gastric (lanes 1-5) and duodenal (lanes 6-9) digestion. Lanes 1-2: BSA controls of gastric digestion; Lanes 3-5: PmPV1 after incubation with pepsin for 0, 60 and 120 min. Lanes 6-7: BSA controls of duodenal digestion; lanes 8-9: PmPV1 digested with trypsin for 0 and 120 min, respectively. (**b**) Other duodenal proteases-120 min digestion. Lanes 1-2: BSA and PmPV1 without enzyme; lanes 3-4: BSA and PmPV1 with elastase (E), respectively; lines 5-6: BSA and PmPV1 with α-Chymotrypsin (α-Ch), respectively; lanes 7-8: BSA and PmPV1 with an Elastase, α-Chymotrypsin and Trypsin (T) mixture. BSA in lanes 3, 5 and 7 were used as positive control. (**c**) Pancreatin digestion. Lane 1: PmPV1 without pancreatin; lanes 2-4 PmPV1 with pancreatin immediately added, 60 and 120 min, respectively; lanes 5-7 pancreatin without PmPV1 at 0, 60 and 120 min, respectively; lanes 8-9: positive (BSA + pancreatin) and negative (BSA without pancreatin) controls after 120 min incubation. (**d**), Proteinase K digestion of PmPV1. Lane 1: Negative control; lines 2-4 BSA with 0, 1, 10, 100 µg/mL proteinase K; lanes 6-9: PmPV1 with 0, 1, 10, 100 µg/mL proteinase K. Lane 5 (std): molecular weight markers of 66, 45, 30, 22.1 and 14.4 kDa.

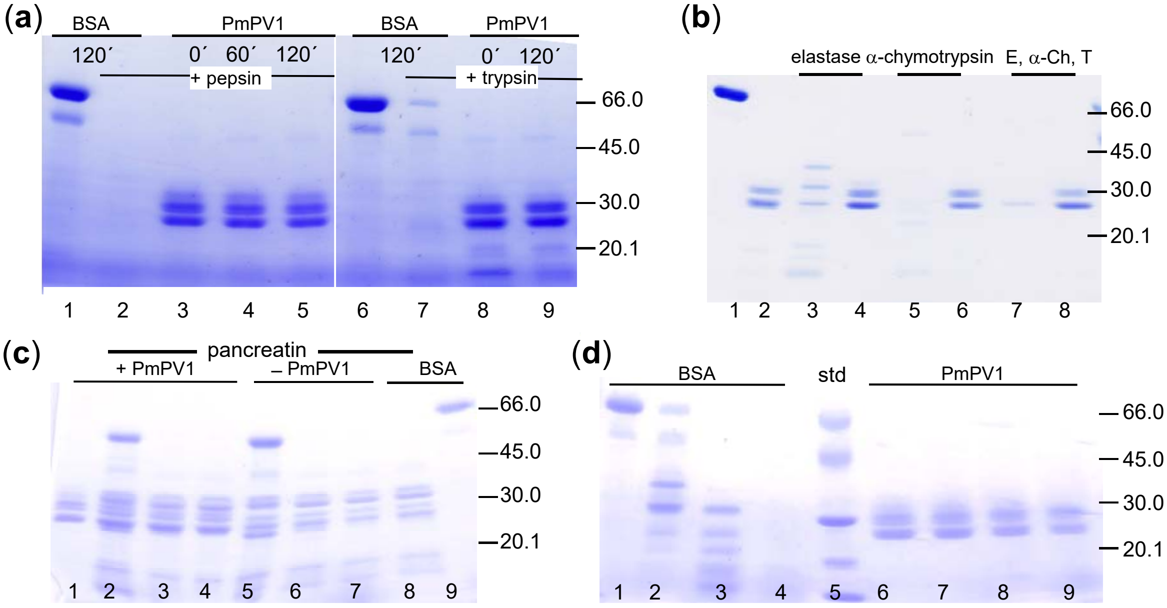

Supplementary Results

Supplementary Figure 4 | *In vivo* digestibility of PmPV1 (**a**) Native PAGE without staining, showing coloured PmPV1; (**b**), Coomassie staining of the same gel shown on (a). (**c**) Western blot of faecal protein antibody known to cross-react with PmPV1 subunits. *i*. Purified PmPV1. *ii*. Faecal proteins showing PmPV1 in faeces collected 1–24 h after first administration. Ctrl: Faecal proteins collected in the control mouse 4 h after beginning the experiment. *iii*. Comparison of PmPV1 with (+GI) or without (-GI) passage through the gastrointestinal tract. Molecular weight marker (std): thyroglobulin (669 kDa), ferritin (yellowish-coloured, 440 kDa), catalase (232 kDa), lactate dehydrogenase (140 kDa), and Albumin (67 kDa).

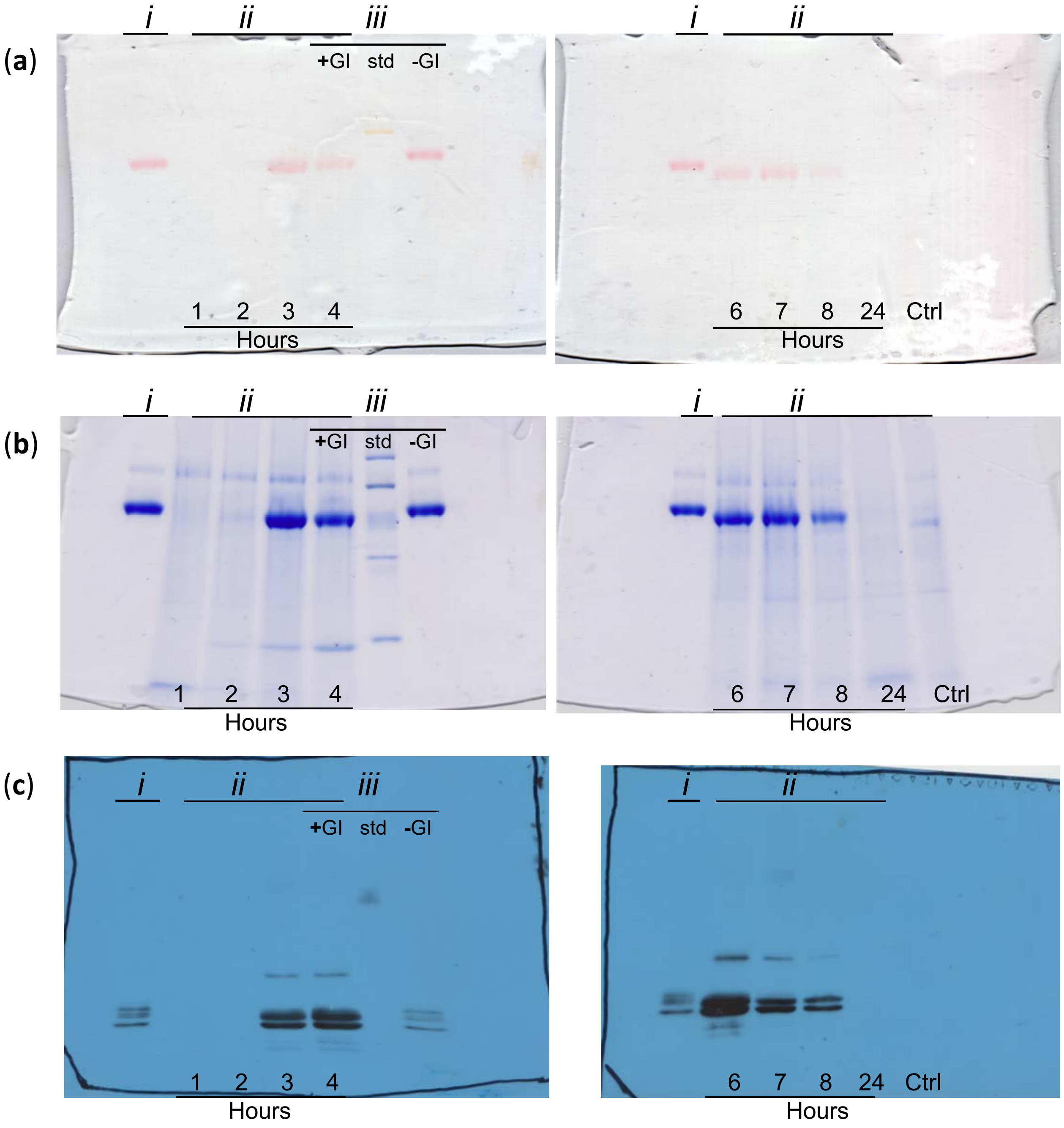

Supplementary Results

Supplementary Figure 5 | Pepsin and trypsin inhibition capacity of PmPV1. (**a**) SDS-PAGE showing no pepsin inhibition by PmPV1. Lane 1, PmPV1 and BSA without Pepsin (negative control); Lane 2, BSA incubated with pepsin. Lane 3, PmPV1 incubated with pepsin. Lane 4, BSA after pepsin incubation for 10 min in the presence of PmPV1. (**b**) Trypsin specific activity without (-PmPV1) and with PmPV1 (+ PmPV1). Ns: not significant (*P>0.05).*

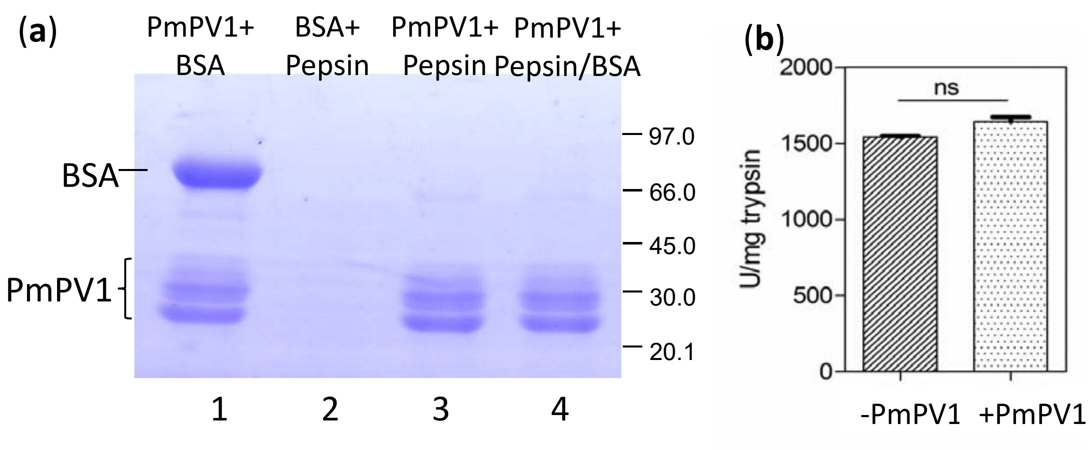

Supplementary Results

Supplementary Figure 6 | Behaviour of PmPV1 with a reducing agent. Dissociating gel electrophoresis (SDS-PAGE) in 4–20% polyacrylamide gradient. Lane 1: Low molecular weight standards; Lane 2: PmPV1 incubated without β-mercaptoethanol; Lane 3: PmPV1 with reducing agent.

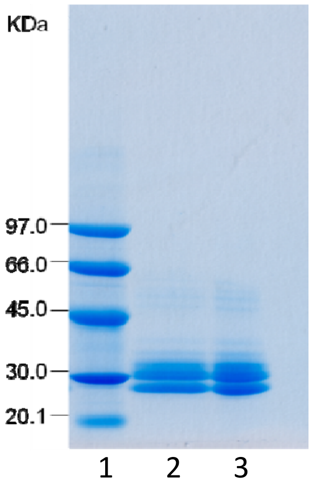

Supplement: Supplementary file 1 — Supplementary Figures and a Table [file 41598_2017_16185_MOESM1_ESM.pdf]
